# One-year experience with ^68^Ga-PSMA PET/CT: applications and results in biochemical recurrence of prostate cancer

**DOI:** 10.1590/0100-3984.2017.0008

**Published:** 2018

**Authors:** Luciano Monteiro Prado Júnior, Fiorella Menegatti Marino, Renato Barra, Leonardo Fonseca Monteiro do Prado, Alaor Barra Sobrinho

**Affiliations:** 1 MD, Specialist in Nuclear Medicine, Attending Physician in Charge of the PET/CT Unit at Imagens Médicas de Brasília (IMEB), Brasília, DF, Brazil.; 2 Doctoral Student in Neuroscience at the Universidade de Brasília (UnB), Attending Physician in the PET/CT Unit at Imagens Médicas de Brasília (IMEB), Brasília, DF, Brazil.; 3 MD, MSc, Attending Physician in the PET/CT Unit at Imagens Médicas de Brasília (IMEB), Brasília, DF, Brazil.; 4 MD, Specialist in Nuclear Medicine, Attending Physician in the PET/CT Unit at Imagens Médicas de Brasília (IMEB), Brasília, DF, Brazil.; 5 MD, Specialist in Nuclear Medicine, Medical Director at Imagens Médicas de Brasília (IMEB), Brasília, DF, Brazil.

**Keywords:** Prostate cancer, Kidney cancer, PET/CT, ^68^Ga-PSMA

## Abstract

**Objective:**

To show the initial (first-year) experience with ^68^Ga-PSMA PET/CT at a clinic in Brazil.

**Materials and Methods:**

Over a one-year period, 96 examinations with ^68^Ga-PSMA PET/CT (85 related to prostate cancer and 11 related to kidney cancer) were performed in 90 patients.

**Results:**

In the prostate and kidney cancer patients alike, the main clinical indication for ^68^Ga-PSMA PET/CT was suspicion of recurrence during follow-up (in 65.8% and 63.0% of the cases, respectively). Among the prostate cancer patients, 38.5% of those with a prostate specific antigen (PSA) < 0.5 ng/mL tested positive for recurrence on ^68^Ga-PSMA PET/CT, compared with 71.0% of those with a PSA of 0.5-0.99, 85.7% of those with a PSA of 1.0-1.99, and 92.6% of those with a PSA > 1.99.

**Conclusion:**

Although ^68^Ga-PSMA PET/CT is a technique that has only recently been applied in clinical settings, despite its high cost, ^68^Ga-PSMA PET/CT shows great promise as a tool in the clinical management of patients with kidney and prostate cancer, especially in those with prostate cancer whose PSA levels are elevated even after treatment.

## Abstract (pt)

**Objetivo:**

Mostrar a experiência inicial de exames de PET/CT com ^68^Ga-PSMA em uma clínica brasileira durante um ano.

**Materiais e Métodos:**

No período de um ano foram realizados 96 exames de PET/CT com ^68^Ga-PSMA, sendo 85 relacionados ao câncer de próstata e 11 relacionados ao câncer de rim, com o envolvimento de 90 pacientes.

**Resultados:**

Tanto no câncer prostático como no câncer renal, a principal indicação clínica foi suspeita de recidiva durante acompanhamento (65,8% e 63,0%, respectivamente). Nos casos de câncer de próstata, os exames foram positivos em 38,5% com o antígeno específico da próstata (PSA) menor que 0,5 ng/mL, em 71,0% com o PSA entre 0,5 e 0,99, em 85,7% com o PSA entre 1,0 e 1,99, e em 92,6% com o PSA maior que 1,99.

**Conclusão:**

O exame de PET/CT com ^68^Ga-PSMA, embora seja uma técnica de aplicação clínica recente e de custo elevado, é uma ferramenta bastante promissora no manuseio clínico de pacientes com câncer de rim e de próstata, principalmente para os casos de câncer de próstata já tratados e que apresentam elevação do PSA.

## INTRODUCTION

A new modality of diagnostic investigation in oncology is ^68^Ga-PSMA PET/CT, which is mainly used in patients with prostate cancer^(^^1^^)^. However, there have been reports of its use in patients with thyroid cancer^(^^2^^)^, breast cancer^(^^3^^)^, kidney cancer^(^^4^^,^^5^^)^, and other types of cancer^(^^6^^)^. Although the physiological process for this application was described several years ago^(^^6^^)^, its use has been validated only in recent studies, most of which were conducted in Europe. In Brazil, the first ^68^Ga-PSMA PET/CT examinations were conducted in 2015. Here, we attempt to study and present our experience with this new diagnostic method at the end of the first year after it had been introduced into practice at our facility.

The objective of this study was to perform a retrospective analysis of the initial (first-year) experience with ^68^Ga-PSMA PET/CT at our facility and to determine whether the results are in agreement with those reported in the main studies in the international literature.

## MATERIALS AND METHODS

A total of 96 examinations with ^68^Ga-PSMA PET/CT were performed between October 7, 2015 and October 6, 2016. Of those 96 examinations, involving a total of 90 patients (4 women and 86 men), 85 were related to prostate cancer and 11 were related to kidney cancer. The mean age of the patients with prostate cancer was 61.5 years (range, 42-94 years), whereas that of the patients with kidney cancer was 59.4 years (range, 21-72 years). All patients were interviewed during the pre-examination orientation session, at which time the patients gave written informed consent and the reason for the examination was discussed with the requesting physician.

Patients were divided into four distinct groups regarding the study objective: 1 - patients referred for diagnostic purposes, for example, when the prostate specific antigen (PSA) was elevated without previous biopsy or when there was a renal nodule to be clarified; 2 - patients who had already received a definitive diagnosis and in whom staging of the disease was required; 3 - patients undergoing the examination for the purpose of evaluating the response to treatment; 4 - patients who had already completed treatment but were under suspicion of recurrence and in whom restaging of the disease was therefore required.

The exams were performed in a 128-slice PET/CT scanner (Discovery PET/CT 710; GE Healthcare, Milwaukee, WI, USA). The protocol was based on those cited in previous studies and was defined by the four nuclear physicians responsible for the PET/CT reports, all with at least four years of experience with ^18^FDG PET/CT reports, the ^68^Ga-PSMA PET/CT reports being produced by at least two physicians (one nuclear physician and one radiologist). The dose administered to each patient was approximately 1.85 MBq (0.05 mCi/kg). The use of contrast for CT was at the discretion of the attending physician and was used in most studies if there was no contraindication. The CT was performed with low-dose protocol (120 kV, 30 mA). The first image, which comprised a scan from the head to the top of the thigh, was acquired 45-60 min after injection of the radiotracer. If there was no contraindication, an intravenous diuretic was administered and a complementary late image of the area(s) of interest, mainly the pelvis, was acquired. The time per bed position was 2.5-4 min, depending on the weight of the patient.

## RESULTS

We performed a total of 96 ^68^Ga-PSMA PET/CT examinations, of which 85 were related to prostate cancer and 11 were related to kidney cancer. Of the 85 examinations related to prostate cancer (in 81 patients), 56 were performed in previously treated patients who were under suspicion of recurrence because of an elevated PSA level; 17 were in patients who had recently been diagnosed with the disease and underwent the examination in an attempt to improve the staging; 7 were in patients who had not yet received a definitive diagnosis but were under strong clinical suspicion, mainly because of an elevated PSA level; and 5 were in patients who had previously undergone ^68^Ga-PSMA PET/CT (4 at our facility and 1 at another facility) and had returned for evaluation of the response to treatment. Of the 9 patients with kidney cancer (11 examinations), 7 patients were in follow-up treatment and under suspicion of recurrence, 2 of the 7 repeating the examination 6 months later (one as a follow-up and the other for evaluation of the response to treatment); 1 patient who had not previously undergone PET underwent the examination for evaluation of the response to treatment; and 1 patient underwent the examination for the investigation of a renal nodule and abdominal lymph node enlargement.

Of the 56 patients who underwent the examination because there was biochemical evidence of recurrence (an elevated PSA level), 2 were excluded from our analysis because they did not bring the report showing their PSA level. Among the remaining 54 patients, the PSA level ranged from 0.02 to 39.0 ng/mL: 0.02-0.49 in 13 patients; 0.50-0.99 in 7; 1.00-1.99 in 7; and > 1.99 in 27. Table 1 shows the ^68^Ga-PSMA PET/CT findings for each patient, by PSA level. Those patients had already undergone CT or MRI of the pelvis and abdomen, as well as bone scintigraphy, as indicated in the main prostate cancer guidelines, prior to undergoing ^68^Ga-PSMA PET/CT, and the results of those previous scans had been negative or inconclusive, given that multiparametric MRI is the best diagnostic method for the evaluation of local and locoregional recurrence^(^^7^^)^. We found that higher PSA levels translated to a higher rate of positivity on the examination and, in general, greater tumor volume. The rate of positivity on the examination was 38.5% for PSA values of 0.02-0.49 ng/mL, 71.0% for PSA values of 0.50-0.99, 85.7% for PSA values of 1.00-1.99, and 92.6% for PSA values > 1.99. The PSA doubling time and the Gleason score could not be assessed, because much of this information was not well understood by the patient or by the attending physician.

**Table 1:** Imaging findings for each patient, by PSA level.

PSA (ng/mL)	^68^Ga-PSMA PET/CT finding
0.02	Negative
0.18	Negative
0.19	Left internal iliac lymph node
0.19	Negative
0.22	Abdominal lymph nodes
0.30	Negative
0.31	Negative
0.32	Prostatic space
0.34	Negative
0.34	Negative
0.36	Presacral lymph node
0.39	Negative
0.40	Liver and bone metastases
0.56	Negative
0.57	Negative
0.68	Pelvic and abdominal lymph nodes
0.70	Prostatic space + pelvic lymph nodes
0.75	Rectovesical nodules
0.96	Pelvic lymph node
0.99	Pelvic lymph node + rib
1.02	Bone metastasis
1.06	Recurrence in the urinary bladder
1.09	Prostate
1.35	Pelvic lymph nodes
1.41	Mediastinal and abdominal lymph nodes
1.65	Negative
1.73	Seminal vesicle and obturator lymph node
2.09	Prostatic space
2.70	Prostatic space
2.84	Obturator region
2.90	Prostate
3.00	Prostate
3.18	Prostate
3.23	Seminal vesicle
3.30	Prostatic space and abdominal lymph nodes
3.61	Negative
3.80	Neurovascular bundle
4.35	Prostate and neurovascular bundle
4.61	Negative
4.61	Abdominal lymph nodes
5.56	Bone/lymph node/lung metastases
5.70	Bone metastasis
6.70	Pelvic and abdominal lymph nodes
6.77	Prostate and seminal vesicle
7.00	Abdominal lymph nodes
8.71	Prostatic space, lung, and bone
11.6	Bone metastasis
13.0	Mediastinal, abdominal, and pelvic lymph nodes
13.4	Inguinal lymph node
13.8	Prostate
15.0	Abdominal lymph nodes
15.0	Bone metastasis
24.3	Metastases to the liver, peritoneum, and thoracic/abdominal lymph nodes
39.0	Multiple lesions of the bone, thoracic/abdominal lymph nodes, pelvis and ureters

Regarding the treatments performed before the examination, 26 patients had undergone prostatectomy only; 7 had undergone radiation therapy; 6 had undergone prostatectomy and radiotherapy; 6 had undergone prostatectomy, radiation therapy, and hormone therapy; 4 had undergone brachytherapy; 2 had undergone radiation therapy and hormone therapy; 2 had undergone prostatectomy, hormone therapy, and chemotherapy; and 1 had undergone prostatectomy, chemotherapy, and radiotherapy. Therefore, 41 (76%) of the 54 patients had undergone prostatectomy.

**Figure 1:**
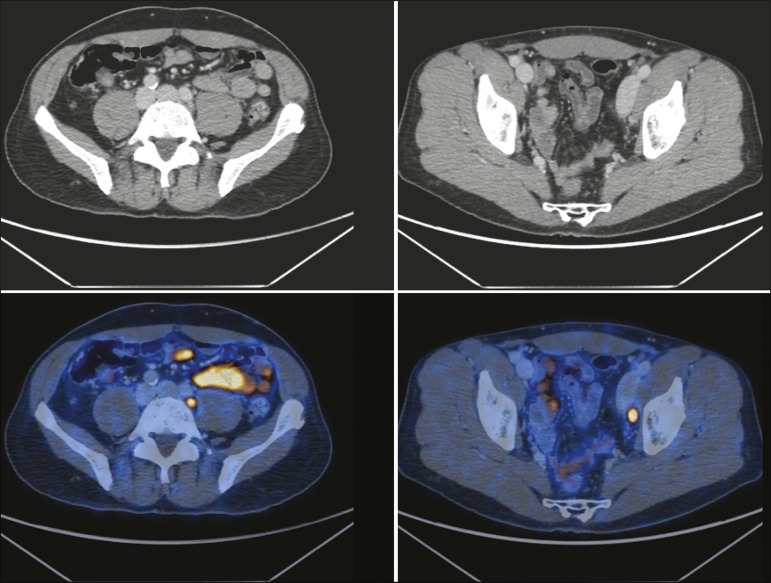
^68^Ga-PSMA PET/CT showing high uptake of the radiopharmaceutical in pelvic, obturator, and left external iliac lymph nodes, which were confirmed as being metastatic.

Most of the patients evaluated were in follow-up treatment. In some cases, histological confirmation was achieved after resection of the lesion. That was the case for a 54-year-old patient diagnosed with prostate cancer 6 years prior, with a Gleason score of 6 (3 + 3), who was treated with brachytherapy and whose most recent PSA levels were 0.20 ng/mL at 2 years prior, 0.68 at 1 year prior, 1.05 at 3 months prior, and 1.35 at the time of the examination. In that same patient, recent bone scintigraphy results were normal, as were those of recent CT scans of the chest and abdomen. The ^68^Ga-PSMA PET/CT examination of that patient showed high uptake of the radiopharmaceuticals in the left pelvic, obturator, and left iliac lymph nodes, which, after resection, were confirmed as being metastatic (Figure 1).

For most of the patients, there was no histopathological confirmation of the positive ^68^Ga-PSMA PET/CT findings. Therefore, we cannot be sure that those patients were truly positive, the indication to make this confirmation being defined by the attending physician and based on the anamnesis of the patient. The clinical evolution will indicate whether the positivity determined by the examination was correct.

## DISCUSSION

The main indication for a ^68^Ga-PSMA PET/CT examination at our facility was suspicion of recurrence after treatment, which was the indication in 56 (58.3%) of the 96 cases, as well as being the first and main indication for this examination reported in the literature. Of the 54 patients with prostate cancer and evidence of laboratory recurrence (elevated PSA level), the majority (76%) had undergone prostatectomy at the beginning of treatment. The rate of positivity on our examinations in relation to the PSA level was similar to that reported in other studies^(^^8^^-^^10^^)^, being even superior to studies with choline (^11^C-choline and ^18^F-fluoromethylcholine)^(^^11^^,^^12^^)^and fluciclovine-18F (anti-1-amino-3-[^18^F]fluorocyclobutane-1-carboxylic acid)^(^^13^^)^. We felt that it would not be appropriate to compare our ^68^Ga-PSMA PET/CT examinations with other previously performed diagnostic tests that produced negative or inconclusive results, because those tests were performed at several other facilities, where different techniques and protocols are employed.

The importance of defining the site of recurrence of the disease is of singular importance in the management of cases. In addition, a ^68^Ga-PSMA PET/CT scan can offer patients with positive results and multiple metastases that are unresponsive to conventional treatments the possibility of treatment with ^177^Lu-PSMA-617 or ^225^Ac-PSMA, which has recently (in the last 2 years) been shown to be safe and efficient^(^^14^^,^^15^^)^.

Although the role of ^68^Ga-PSMA PET/CT in the staging of prostate cancer has yet to be well defined, some studies have already shown its superiority in relation to tests routinely performed for this purpose, such as bone scintigraphy^(^^16^^)^, which rarely adds any information to that obtained with ^68^Ga-PSMA PET/CT, and recent studies have suggested that the latter is a good option in high-risk patients; that is, those with a Gleason score ≥ 7 (4 + 3) and a PSA level > 10 ng/mL^(^^17^^)^. Of the 96 examinations in our sample, 17 (17.7%) were performed for that purpose (staging). However, the real value of this image modality will become clear only during the follow-up of these patients and in future studies.

The other applications for which ^68^Ga-PSMA PET/CT is employed at our facility will also need to be better defined in the future, although some small cases series have been conducted, for example, for the evaluation of the response to treatment of kidney and prostate cancer^(^^18^^,^^19^^)^, suspicion of recurrence of kidney cancer^(^^4^^)^, and mapping of the extent of disease in the prostate^(^^20^^)^.

## CONCLUSION

Because it is a fairly new technique (not yet included in the main oncology guidelines), ^68^Ga-PSMA PET/CT is restricted to a few diagnostic imaging centers and has a relatively high cost. Consequently, it has been difficult to incorporate the method into routine clinical practice at oncology centers. However, many studies have shown that it has excellent accuracy in localizing prostate cancer recurrence and changes in the behavior of the disease^(^^8^^-^^11^^)^, with results similar to those presented in our study. Nevertheless, given the incipient nature of this technique, its impact on the overall survival of these patients can be defined only in the future.

Other applications of ^68^Ga-PSMA PET/CT will require further study. However, it is already evident that, in certain clinical contexts, such as the staging of prostate cancer^(^^16^^,^^17^^)^and the suspicion of recurrence of kidney cancer^(^^4^^)^, this method, if applied properly, can facilitate the work of the oncologist. The initial results obtained through the application of ^68^Ga-PSMA PET/CT at our facility have been quite satisfactory and encouraging.
